# Isolated Tuberculosis of the First Metatarsal of the Left Foot With Congenital Hallux Valgus Without Pulmonary Involvement: A First of Its Type Case

**DOI:** 10.7759/cureus.41008

**Published:** 2023-06-26

**Authors:** Sankalp Yadav, Gautam Rawal, Madhan Jeyaraman

**Affiliations:** 1 Medicine, Shri Madan Lal Khurana Chest Clinic, New Delhi, IND; 2 Respiratory Medicine, Max Super Speciality Hospital, New Delhi, IND; 3 Orthopedics, South Texas Orthopedic Research Institute (STORI) Inc., Laredo, USA; 4 Biotechnology, School of Engineering and Technology, Sharda University, Greater Noida, IND; 5 Regenerative Medicine, Indian Stem Cell Study Group (ISCSG) Association, Lucknow, IND; 6 Regenerative Medicine, Datta Meghe Institute of Higher Education and Research, Wardha, IND; 7 Orthopedics, ACS Medical College and Hospital, Dr. MGR Educational and Research Institute, Chennai, IND

**Keywords:** mycobacterium tuberculosis, skeletal tuberculosis, tuberculosis, hallux valgus, first metatarsal

## Abstract

Osteomyelitis caused by *Mycobacterium tuberculosis* is a rare entity. This clinical condition is even rarer when the bones of extremities like the foot are involved. There is a paucity of literature on the isolated tubercular involvement of the bones of the foot. Further, congenital hallux valgus is an infrequently reported condition. A case of tuberculosis of the first metatarsal of the left foot with congenital hallux valgus without pulmonary involvement is never reported in the literature. We herein present a first of its type case where tuberculous osteomyelitis of the first metatarsal of the left foot with congenital hallux valgus was diagnosed with the help of radiology, cartridge-based nucleic acid amplification test, and pus culture. The patient was initiated on anti-tubercular treatment but was ultimately lost to follow-up.

## Introduction

Tuberculosis is a major public health issue, especially in low-income countries in Asia, Africa, and Europe [1]. The disease is known from ancient times, and the latest statistics from the year 2021 show an incidence of 188 and a prevalence of 312 per one lakh (0.1 million) population in India [2].

Compared to pulmonary cases, extrapulmonary tuberculosis is infrequently reported (10-15% of total tuberculosis cases) [3]. Skeletal tuberculosis accounts for one to three percent of all extrapulmonary cases and involvement of bones of the foot is unwonted [4]. The bones of the foot commonly involved are calcaneum, talus, first metatarsal, navicular, and medial and intermediate cuneiforms [5]. The incidence of osteomyelitis of the metatarsal is <0.5% [6]. The commonly involved metatarsals are the first and fifth [7].

As compared to adults and adolescents, where hallux valgus is a frequently reported deformity, congenital hallux valgus is very rare [8]. There is a scarcity of knowledge related to this deformity and its cause [8]. In a study from 1984 to 1987 on 5700 newborns, only eight cases were reported [9]. Further, there are discrepancies about the etiology of this deformity in the medical literature [8].

Isolated tubercular involvement of bones of the foot without pulmonary involvement is seldom reported and the case becomes extremely rare when the patient has congenital hallux valgus deformity. The present case is a case where tuberculosis of the first metatarsal of the left foot with congenital hallux valgus without pulmonary involvement is reported in an eight-year-old Indian female. The diagnosis was established with the help of radiology, cartridge-based nucleic acid amplification test, and pus culture. The patient was initiated on anti-tubercular treatment for 12 months.

## Case presentation

An eight-year-old Indian female child belonging to low-socioeconomic background was brought by her parents to the outpatient department with complaints of pain and wound over the plantar aspect of the left foot for six months. It was associated with purulent (yellow-colored, non-foul-smelling) discharge for three months. She was not able to bear weight on the left foot and this was associated with a limp.

The pain was insidious in onset and gradually progressed with the development of the wound. It was continuous, localized on the left foot, and aggravated on walking. Her pain subsided slightly post-consumption of over-the-counter non-steroidal anti-inflammatory drugs (NSAIDs). There was no history of night sweats, fever, cough, or weight loss. And there was no history of trauma. Besides, there was no history of tuberculosis in her, her family, or any contacts. Her past history was remarkable for congenital bilateral hallux valgus for which no major treatment was done. Besides, the parents were questioned in detail about a possible child abuse due to a delayed consultation; however, they attributed the delay in reporting to the lockdowns due to the coronavirus disease 2019 (COVID-19) pandemic.

**Figure 1:**
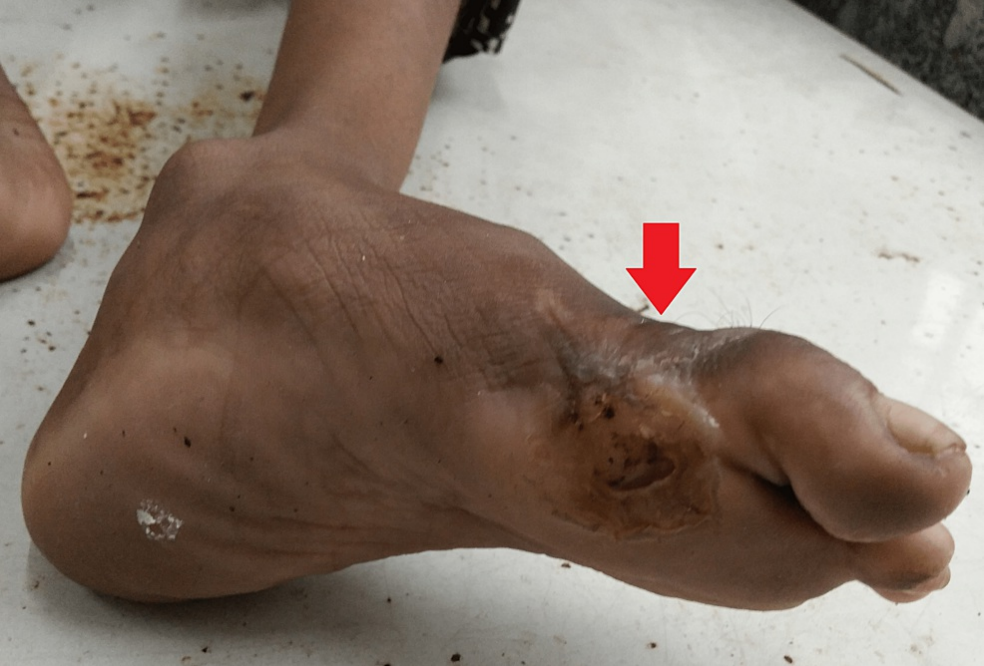
Wound with active purulent discharging sinus over the plantar aspect of the left foot.

**Figure 2:**
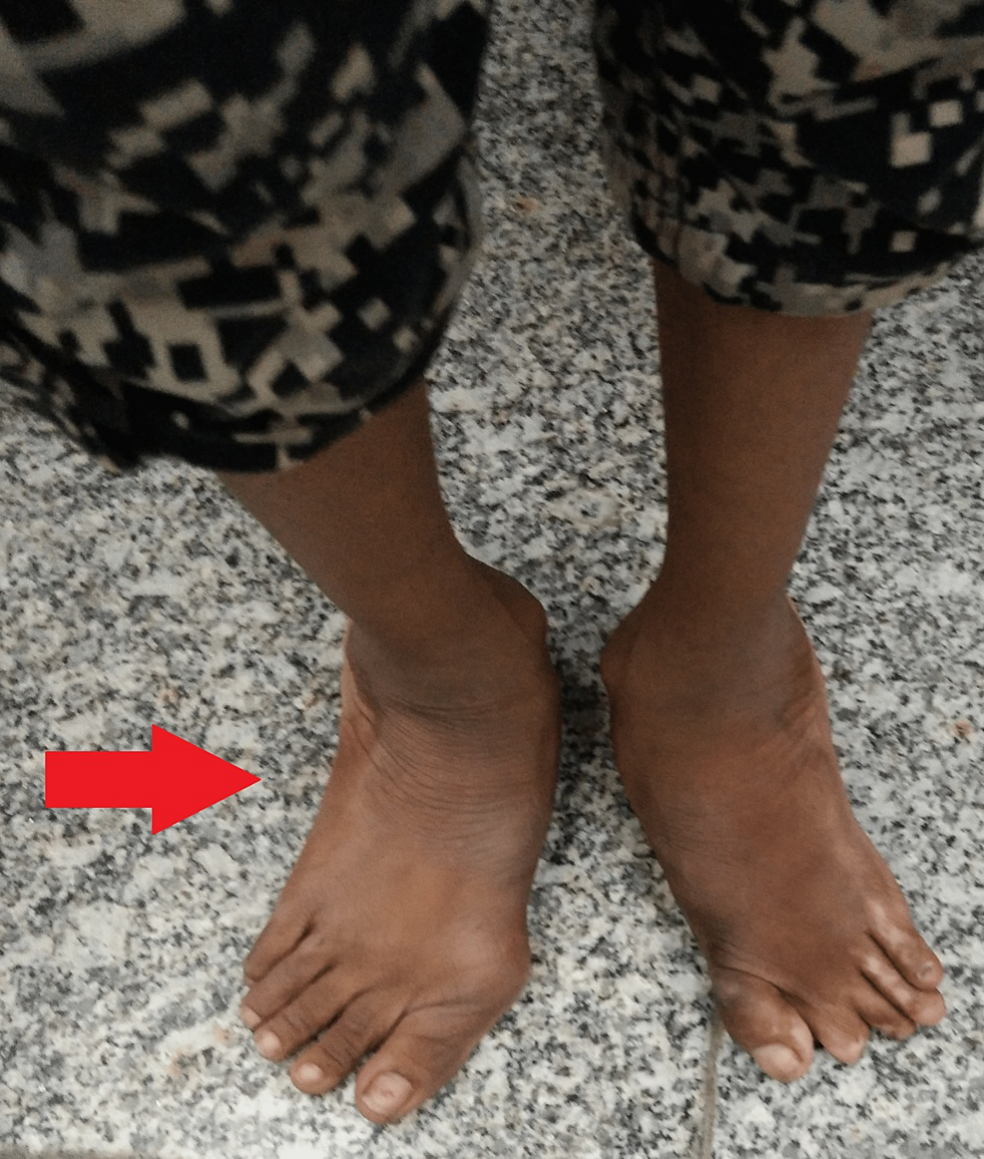
Gross image of bilateral hallux valgus.

General examination revealed a young child with a temperature of 98.4°F, pulse of beats 79 per minute, blood pressure of 110/80 mmHg, respiratory rate of 18 breaths per minute, and oxygen saturation (SpO2) of 99% on room air. Local examination revealed a 4×5 cm wound with an active purulent discharging sinus over the plantar aspect of the left foot over the first metatarsal with diffuse edges (Figure 1). There was a limited range of movement of the first metatarsophalangeal joint due to pain. The eversion and inversion movements of the left foot were comparatively limited and painful while dorsiflexion and plantar flexion were terminally painful. The adjoining skin around the sinus was warm to the touch and erythematous but there were no dilated veins. Further, there was marked bilateral hallux valgus deformity (Figure 2). Other joints of the left foot were normal. And there were no similar findings on the right foot except for the hallux valgus deformity resulting in reduced first metatarsophalangeal joint range of movement due to pain and reduced first tarsometatarsal mobility. Further, there was no koilonychia, clubbing, icterus, cyanosis, pallor, or lymphadenopathy. Her systemic examination was within normal limits.

Based on non-specific clinical features, the high burden of tuberculosis in the country, and the initial examination, a preliminary diagnosis of tuberculosis of the left foot was made with differentials fungal osteomyelitis, pyogenic osteomyelitis, bone tumor, and granulomatous diseases such as gout, amyloidosis, and sarcoidosis.

All the serological indicators were within normal limits except a raised erythrocyte sedimentation rate of 55 mm in the first hour, her induced sputum for acid-fast bacilli and cartridge-based nucleic acid amplification test (CBNAAT) were negative. Also, her HIV was non-reactive. Her chest radiograph was unremarkable.

**Figure 3:**
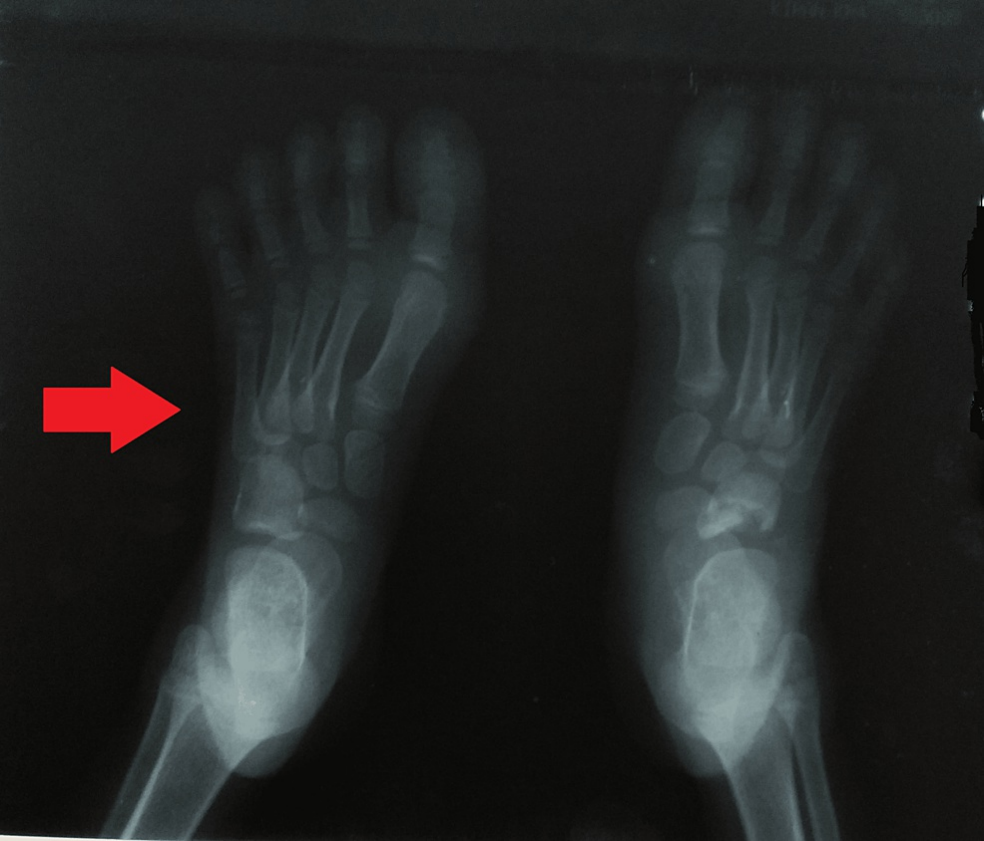
Radiograph of the feet revealed bilateral hallux valgus deformity.

**Figure 4:**
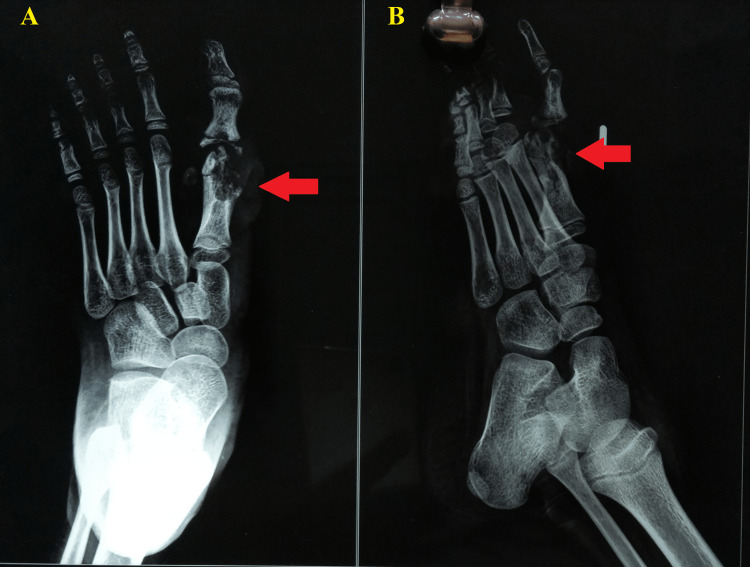
Anteroposterior (A) and oblique (B) radiograph of the left foot.

An old plain radiograph of both feet revealed bilateral hallux valgus deformity (Figure 3). An anteroposterior and oblique radiograph of the left foot was suggestive of an expansile osteolytic lesion with cortical thinning of the first metatarsal head (Figures 4A, 4B). Additionally, a magnetic resonance imaging of the foot was suggestive of a lytic lesion measuring 16×19 mm involving the head and distal shaft of the first metatarsal and a bony sequestrum connecting to the plantar surface with a sinus tract.

The patient underwent an open biopsy and wound debridement. About 20 mL of yellowish-colored pus was drained. The medullary cavity was thoroughly curetted, sinus tracts were excised, and the samples were sent for histopathological and microbiological investigations.

Ziehl-Neelsen staining for acid-fast bacilli from the aspirated pus was negative. Histopathology was indicative of tuberculosis with epitheloid granulomas with caseating necrosis and lymphocytes. The CBNAAT of the pus revealed *Mycobacterium tuberculosis* detected (low) with no resistance to rifampicin. An additional sample was sent for line probe assay (LPA) and culture to the National Reference Laboratory and the results were suggestive of *M. tuberculosis* detected on LPA and grew on liquid culture media with no resistance to any of the first-line drugs for anti-tubercular treatment.

Finally, a diagnosis of tuberculosis of the first metatarsal of the left foot with bilateral congenital hallux valgus without pulmonary involvement was made, and she was put on anti-tubercular treatment per her weight initially with four drugs (56 doses) and followed with three drugs for a period of 10 months (Table 1). Along with this she was given a tablet of pyridoxine 1 mg/kg/day for the full course duration and was given dietary advice for a high protein diet. An orthopedician was consulted for her hallux valgus who decided to continue with non-operative management until the closure of physis and advised shoe modifications, besides her parents were reluctant to any surgery.

**Table 1:** Anti-tubercular treatment according to her weight.

Phase	Drug	Dose	Duration
Intensive phase	Rifampicin	10 mg/kg	Eight weeks
	Pyrazinamide	25 mg/kg	Eight weeks
	Ethambutol	15 mg/kg	Eight weeks
	Isoniazid	5 mg/kg	Eight weeks
Continuation phase	Rifampicin	10 mg/kg	Forty weeks
	Ethambutol	15 mg/kg	Forty weeks
	Isoniazid	5 mg/kg	Forty weeks

For the first two months, she responded well to the anti-tubercular treatment with no major adverse drug events, and at the request of her parents, she was transferred out to her village. All the possible efforts were done to contact her after one year but we could not retrieve her recent radiographs.

## Discussion

Tuberculosis is a big burden on public health [1]. Disease contributes to a very high number of morbidity and mortality [2]. Osteoarticular tuberculosis contributes to less than 3% of all cases of extrapulmonary tuberculosis and it can involve joints, bones, tendons, and bursae [10]. Tuberculosis of bones of the foot like metatarsals is less frequently reported and isolated cases without pulmonary involvement in immunocompetent individuals are scarce [11].

Diagnosis of tuberculosis of the bones of the foot is taxing [12,13]. Often the cases are missed due to similarities of this clinical presentation with other musculoskeletal disorders [12]. The common presentations are non-specific, such as stiffness, pain, and swelling [13]. Moreover, the paucibacillary nature of the disease is an important contributor, as until the use of radiometric investigations a definite diagnosis cannot be established [12,13]. Another factor for the diagnostic delay is inadequate awareness among the treating clinicians [14]. The present case is a good example where a patient with congenital hallux valgus was diagnosed after a detailed lab workup supported by radiography and magnetic resonance imaging.

A case sharing similarities with ours was presented by Hennawi and Al-Ahmari in a pediatric patient [11]. The present case shares similarities with their case in gender, absence of constitutional symptoms, clinical findings, radiography findings, and detection of *M. tuberculosis *[11]. However, their case differed from ours in the absence of trauma, no family history of tuberculosis, bilateral hallux valgus deformity, and discharging sinus at the plantar surface.

One more similar case was presented by Sarwal et al. which shares similarities with the present case in no pulmonary involvement, lack of constitutional symptoms, clinical presentations like swelling and presence of discharging sinus, raised erythrocyte sedimentation rate, radiological and histopathological findings [15]. However, it differed from our case due to the presence of family history and no detection of *M.* *tuberculosis *[15].

Another case of a 19-year-old boy similar to ours was published by Madi et al. [6]. Our patient shares similarities with their case in the absence of constitutional symptoms, clinical presentation, the presence of discharging sinus, involvement of first metatarsal bone, histopathological findings, absence of any trauma, and no pulmonary involvement [6]. However, the present case has a few unique features like detection of *M. tuberculosis* on CBNAAT, LPA, and liquid culture, bilateral hallux valgus deformity, different locations of discharging sinus, gender, and age.

The management of the tuberculosis of bones of the foot like metatarsals is essentially medical and involves the use of anti-tubercular chemotherapy [16]. The same is detailed in the National Tuberculosis Elimination Program (India) guidelines [16]. In advanced disease with joint destruction surgical interventions are indicated like biopsy, debridement, synovectomy, distraction, and arthrodesis [13].

This case is an important addition to the literature due to paucity of the data related to metatarsal tuberculosis in the absence of pulmonary involvement. It is also emphasized that such cases should be reported, especially from the high-burden countries as it will increase the awareness among the treating clinicians.

## Conclusions

Isolated tuberculosis of the metatarsal bones of the foot is a rare clinical occurrence. Often these cases are reported late due to the rarity of the condition, a benign beginning, and similarities with other conditions which ultimately impact the treatment outcomes adversely. A very high degree of suspicion with prompt diagnosis using the biopsy and radiographic investigations is imperative for starting the treatment.
